# Exploring the Impact of Leadership Characteristics on Subordinates’ Counterproductive Work Behavior: From the Organizational Cultural Psychology Perspective

**DOI:** 10.3389/fpsyg.2022.818509

**Published:** 2022-02-18

**Authors:** Yaoping Shen, Xinghui Lei

**Affiliations:** School of Economics and Management, Tongji University, Shanghai, China

**Keywords:** cognitive differences, cultural psychology, cross culture, counterproductive work behavior, leadership psychological characteristics, leadership ability characteristics, resource perception

## Abstract

Counterproductive work behavior (CWB) is extremely detrimental to an organization and its stakeholders as they impact economic efficiency and damage the atmosphere within the organization. The culture and personality of leaders can affect their behavior, psychology and ability. Leaders are in a position of authority, have resources and decision-making power, and their words and actions are noticed and imitated by employees. From a leadership perspective, an effective way to avoid CWB is to seek ways to reduce in its occurrence and escalation. First, we conducted a grounded theory study on the leadership characteristics which are the antecedent variable of CWB, and the leadership characteristics were divided into three categories: psychological, behavioral, and ability. These characteristics impact subordinates’ CWB. Second, based on the conservation of resource theory, we conducted an ecological validation of the mechanism through which leadership characteristics affect subordinates’ CWB, explored the role of work resources and personal resources in it. The results indicate that all three types of leadership characteristics have a negative effect on subordinates’ CWB, among them, the mediating effect of work resources was established, and the mediating effect of personal resources was established in some cases. Therefore, different characteristics of leaders will affect the cognitive differences of subordinates to resources, and then trigger behavioral responses of subordinates. By an advantage analysis of the three leadership characteristics on subordinates’ CWB, it is found that the three leadership characteristics are of similar importance on interpersonal CWB. However, in the effect of organizational CWB, the characteristics of leadership have obvious advantages.

## Introduction

In recent years, counterproductive work behavior (CWB) become increasingly prevalent in the workplace, and subordinates’ CWB harm corporate interests and negatively affect companies (Van Zyl and De Bruin, 2018; Wei et al., 2019; Seriki et al., 2020; Wurthmann, 2020). Counterproductive work behavior is an umbrella term encompassing similar harmful behaviors at work, such as aggression, transgression, and retaliation (Spector and Fox, 2010). It refers to any behavior that may cause or has caused potential or substantial harm to the legitimate interests and members of an organization, regardless of whether it violates norms (Sackett, 2002; Spector and Fox, 2002; Oboyle et al., 2011), and includes employees chatting on WeChat during work hours, acting without following instructions, complaining about leaders and the company, frequent job hopping, and reimbursement falsification (Wang et al., 2020). Scholars’ growing interest in CWB is not only for theoretical reasons but also due to several public scandals in this century (Spain et al., 2014) and the fact that the uncivil treatment of employees in the workplace has grown over time and is twice as prevalent as 20 years ago (Schilpzand et al., 2016). Studies also indicate that CWB has spread through organizations, severely damaging their reputation, economic performance, and social image, and is a major challenge for organizational behavior management.

Some scholars have studied CWB and summarized the antecedent variables as job traits, organizational factors, employee cognitive factors, and leadership characteristics (Organ and Konovsky, 1989; Podsakoff et al., 2000; Lau et al., 2003; Schulte-Braucks et al., 2019). However, despite several studies on leadership characteristics, including various leaders’ working styles and behaviors, a system does not exist, and leadership characteristics that trigger CWB in subordinates have still not been fully explored. Chinese people have respected those in high positions since ancient times, and leaders have a special status in a company. Thus, their intrinsic values, speeches, behaviors, and work abilities are noticed, imitated, and learned by employees. Negative behaviors of leaders are transmitted through the management hierarchy, causing subordinates to engage in similar behaviors (O’ Fallon and Butterfield, 2012; Robinson et al., 2014). Abusive supervision triggers deviant behaviors (Tepper, 2007), CWB (Mitchell et al., 2015; Ma et al., 2017), and feedback avoidance behaviors in the workplace (Shen and Yang, 2020). In contrast, leaders’ positive characteristics inhibit subordinates’ CWB. For example, pragmatic leaders promote advice-seeking behaviors in employees (Xu et al., 2021), and ethical leaders negatively influence subordinate CWB (Zhang, 2017; Ahmad, 2018) and promote pro-social behaviors (Mao et al., 2020). Optimistic leaders believe in removing barriers to allow employees to achieve their goals through their efforts and encourage employees to show initiative (Brissette et al., 2002). In addition, leaders with outstanding abilities are more capable of stimulating recognition and followership behaviors, promoting teamwork and goal achievement, and reducing CWB (Zhou and Long, 2016).

Previous studies have demonstrated that leadership characteristics affect subordinate CWB, but the underlying mechanisms are still worth exploring. According to the leader–member exchange theory, leaders divide team members into “insiders” and “outsiders.” Insiders will recognize that they have more resources and increase their work input and reduce absenteeism, while outsiders often perceive the unfair distribution and their lack of resources, which leads to negative emotions, less commitment, and an increase in negative behaviors (Dansereau et al., 1975). The conservation of resource (COR) theory suggests that individuals, whether insiders or outsiders, always try to acquire and retain valuable resources and avoid losing them (Hobfoll, 1989, 2002). The basic assumption of the COR theory is that individuals always try to acquire and retain valuable resources to avoid the loss of resources. When resources are threatened or lost, pressure will occur (Hobfoll, 1989). In the face of pressure, individuals are more likely to demonstrate CWB to deal with the loss of resources and retaliate against the organization (Bordia et al., 2008), and thereby, gain resources to compensate for the loss (Spector and Fox, 2005). Previous studies have shown that there is a positive correlation between work stress and CWB (Fox et al., 2001; Penney and Spector, 2005; Yang and Diefendorff, 2009; Meier and Spector, 2013; Eschleman et al., 2015). When the depletion of employees’ resources reaches the edge of their stock of resources, they will have a sense of burnout. In order to preserve the existing resources, they usually do not continue to invest resources at work. Therefore, burnout will lead to decreased performance, absenteeism and other consequences (Shirom, 1989, 2003).

Therefore, a depletion of both personal and work resources is a psychological trigger that leads to CWB. From the resource loss and acquisition perspective, the COR theory has a good explanatory power for examining an individual’s CWB. This study explores the influence of leadership characteristics on subordinates’ CWB, and its mediating mechanisms based on the COR theory.

## Study I: Leadership Characteristics Based on the Grounded Theory

In the past, the description of leadership factors was vague, and there was no clear definition and classification of the connotation of the concept. Previous literature that explored leadership characteristics included various leadership styles—such as ethical leadership and destructive leadership—leadership behaviors—such as leaders’ rejection, leaders’ abusive behavior, and the exchange relationships between leaders and subordinates—and all the antecedent variables of leadership-related CWB as leadership characteristics. However, they have not been organized and their definitions, categories, and dimensional divisions have not been systematically classified. Therefore, the purpose of Study I is to clearly define and divide the connotation and dimensions of leadership characteristics based on the grounded theory, and to explore the relationship between leadership characteristics and subordinates’ CWB and the role of resources perception from a qualitative perspective.

Grounded theory is a qualitative research method (Glaser and Strauss, 2006). It is based on identifying the area to be studied, obtaining new concepts and theories based on relevant information, and bringing to light the “real inner phenomenon.” Therefore, based on grounded theory, Study I constructs the CWB antecedent variable and a new conceptualization of leadership characteristics through data collection, data coding, and theoretical saturation testing.

### Data Collection

The project team collected the data through interviews. Before conducting the interviews, an interview outline was established by reviewing a large body of literature and research as well as soliciting the opinions of experts in the field. The outline is based on four questions (e.g., “How long have you been working for your current company?”) and five formal interview questions (e.g., “What types of leaders do you like?”). Next, in May 2020, we interviewed 33 employees from the Hangzhou LDK Company, who were randomly selected by HR department from the company-wide personnel list. Of which, 32 employees were successfully interviewed (M_age_ = 41.1years, 28% female). The interviewees’ work experience ranged from 5 months to 20 years; their departments included logistics, administration, finance, quality inspection, quality control, and production, and their positions included ordinary employees, basic-level managers, and middle-level managers. The interviews were conducted by three assistant researchers in three separate rooms. After obtaining the interviewees’ consent, the interviews were recorded. The duration of the interviews ranged from 8 to 32 min. Finally, the interview data were summarized and coded.

### Data Coding

#### Open Coding

From the data obtained from the 32 interviewees, keywords were extracted to form the initial concepts. In this study, a total of 303 initial concepts that satisfied the expression of the concepts were extracted, as shown in Table 1.

**TABLE 1:** Open coding examples.

Interviewees	Original interview content	Concept symbol extraction
Mr. G	The leaders can communicate with us on various issues, such as ideological education, or they care about our life. There will be quarrels, but we are all for the work. I must apologize to the leader if it is my fault, or if the leader is wrong, he will say “it is truly my fault.” The leaders should pay attention to the attitude when quarreling. Be calm.	a1. Care for various issues regarding life and mind state. a2. Communication and cooperation. a3. Attitude.
Mr. H	This leader is competent when taking actions. He will put his ideas into practice. When we work overtime, he will too, and sometimes come to work earlier than we do.	a34. The leader has strong hands-on skills. a35. The leader has a high level of initiation. a36. The leader works overtime with us.
Ms. E	He handles affairs in a decisive manner and I appreciate it. We may differ on some issues, and I may sometimes be a little resistant, but he will talk to us until we have talked it through. The leader is affable and easy to speak to.	a91. Handle affairs in a decisive manner. a92. Communication with subordinates. a93. Strong affinity.

#### Axial Coding

Based on the initial concepts obtained using open coding, selective coding was more directed and targeted at filtering the entries to extract subcategories and core categories. The study finally obtained 11 subcategories from the 303 initial concepts extracted using open coding. Further categorization and integration were conducted to isolate five core categories, namely leadership psychological characteristics, behavioral characteristics, ability characteristics, subordinates’ resource perceptions, and behavioral reactions.

#### Selective Coding

The interview data indicate that there is a causal relationship among the five core categories. The three characteristics of leaders can be taken as the antecedent variables of subordinates’ resource perception, which in turn can be used as the antecedent variable of subordinates’ behavioral reactions. The analysis is centered on the following core categories: leadership psychological characteristics, leadership behavioral characteristics, and leadership ability characteristics that influence subordinates’ perceived access to resources in the organization and from leaders, which further triggers different behavioral reactions from subordinates; in addition, subordinates’ direct perception of leaders’ three characteristics also triggers behavioral reactions.

### Theoretical Saturation Test

The conceptual symbols in the response data of the last seven interviewees overlapped with more than 200 conceptual symbols previously gained. No new concepts or connotations emerged, thus proving that the study’s theory was saturated.

### Results

Based on the aforementioned grounded theory study, the results confirm the negative influence relationship between leadership characteristics and subordinates’ CWB, and depicts the mechanism of action of resources in the context of practices.

#### Leadership Characteristics

Based on the grounded theoretical analysis, leadership characteristics refer to all performances of a leader at work, which can be categorized as: psychological, behavioral, and ability characteristics. Psychological characteristics refer to the intrinsic traits and inherent states of leaders, including personal characteristics, attitude toward others, and psychological qualities. Behavioral characteristics refer to the behavioral performance of leaders and are changeable external states of leaders, including positive and negative behaviors. Ability characteristics refer to leaders’ performance and attitude at work, professionalism, management ability, and work attitude.

#### Subordinates’ Perception of Resources

Judging from the interview data, it is clear that subordinates perceive a wide range of resources such as work initiative, self-identity, and fairness in performance evaluations. With regard to the different characteristics of leaders, there are differences in how subordinates perceive the resources they own. When subordinates perceive leaders’ positive characteristics, they also perceive job fairness and freedom, and high job satisfaction, leading to increased self-efficacy. In contrast, when subordinates perceive leaders’ negative characteristics, they have negative evaluations and attitudes, perceive less support and freedom from their leader and team, and less control over their environment, and consequently fewer work and personal resources.

#### Subordinates’ Behavioral Reactions

Different manifestations of leadership characteristics can directly influence subordinates’ behaviors and trigger behavioral reactions by affecting their perception of resources. Negative manifestations of leadership characteristics will trigger a decrease in subordinates’ perceived resources and lead to dissatisfaction, negativity at work, retaliation, and ultimately cause harm to the organization. Positive manifestations allow subordinates to perceive more support, which leads to higher levels of self-esteem and optimism, so that they will work more efficiently and even take initiatives to engage in things that are beneficial to the organization and beyond their job requirements or leaders’ expectations and organizational norms.

#### Relationship Between the Variables

**FIGURE 1:**
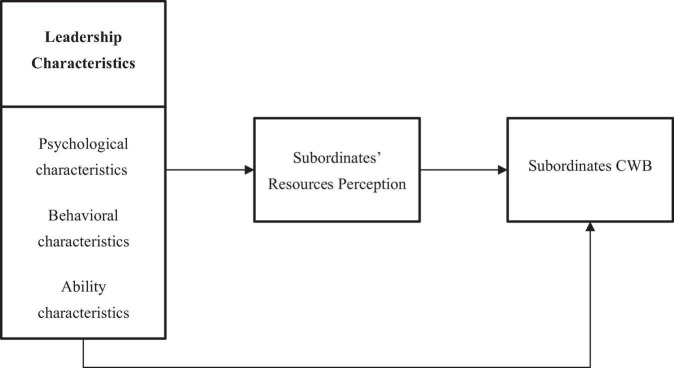
Grounded theory result model.

All three leadership characteristics act directly on subordinate CWB. However, when leadership characteristics do not meet subordinates’ expectations, subordinates’ perceived resources will be damaged. Consequently, subordinates will tend to vent their dissatisfaction to regain resources, and engage in negative behaviors to harm the workplace, their relationship with the leader, and the organization. In other words, subordinate CWB is triggered, and resource perception plays a mediating role. Ultimately, an influence path of subordinate CWB is established based on changes induced by leaders’ behaviors in subordinates’ perceived resources. The grounded theory model is illustrated in Figure 1.

## Study II: Empirical Study on the Influence Mechanism of Leaders’ Multi-Dimensional Characteristics on Subordinate CWB

The second study is based on the COR theory, and uses a quantitative method to explore the internal mechanism of leadership characteristics that influences subordinates’ CWB through the role of resources. The study starts from the perspective of leadership and distinguishes the priorities in the process of avoiding subordinates’ CWB to compare the relative importance of the three characteristics in influencing subordinates’ CWB, and provides theoretical support for corporate practice.

### Theories and Hypotheses

#### Leadership Characteristics and Subordinate CWB

The psychological characteristics of leaders refer to their intrinsic qualities. Leaders with a high level of empathy allow employees to identify themselves as “insiders,” and this perception stimulates positive emotions, intrinsic motivation, a sense of responsibility, and promotes employees’ organizational citizenship behaviors (Liu et al., 2015). In a team context, optimistic leaders believe that through their efforts, they can eliminate the obstacles to achieving their goals, so as to encourage members to work actively (Brissette et al., 2002). Leaders with high emotional intelligence can create a sound interpersonal atmosphere and implement effective motivation measures to increase employees’ satisfaction with the organization (Dasborough and Ashkanasy, 2002). This triggers more positive emotions and leads employees to engage in more proactive behaviors (Fredrickson, 2001). Thus, at work, leaders with positive psychological characteristics motivate employees to exhibit less CWB and increase positive work behaviors, while leaders with negative characteristics cause emotional burnout in subordinates and disrupt the leader–member exchange relationship, which in turn triggers CWB in subordinates. The following hypothesis is proposed:

**Hypothesis 1 (H_1_**): The state of leadership psychological characteristics impacts subordinate CWB.

Due to the special status of leaders, their behaviors will receive more attention and imitation from subordinates. Behavioral leadership theory holds that leaders who care and help their subordinates can stimulate more organizational citizenship behaviors and advice-seeking behaviors among employees. Leaders’ negative behavioral characteristics trigger negative effects. Unfair treatment by leaders will cause employees to develop negative cognitions and emotions, and to feel more inclined to pay greater attention to personal interests (Morrison and Robinson, 1997), ignore organizational interests, and engage in behaviors that harm the organization to gain personal advantage. In addition, strong social interaction is found in CWB, which relies on communication and mutual influence among organizational members and rarely culminates in isolation (Ashforth and Anand, 2003). Many other factors that are closely related to CWB, such as negativity at work (Huang et al., 2016) and unethical behaviors (Umphress et al., 2010), affect subordinate CWB through downward transmission. Most unethical behaviors are not subjective but arise from the behaviors of superiors, such as hints, threats, and inducements (Wang, 2010). The following hypothesis is proposed:

**Hypothesis 2 (H_2_):** The state of leadership behavioral characteristics impacts subordinate CWB.

Leaders make up the core talents of a company and serve as role models. Their abilities reflect the comprehensive qualities associated with being a leader and affects observers’ psychological perceptions of leaders and observers’ behavior (Zhou and Long, 2016). Leaders’ abilities have a significant positive effect on the performance behaviors of individual employees (Borman, 1993; Conway, 1999). It determines employees’ perception of their leaders’ credibility, influences employees’ own behavior, and is a necessary factor for leaders to build trusting, reliable, and effective relationships with employees (Boyce et al., 2010). Therefore, greater ability will help leaders win greater trust from employees, and employees will be more willing to engage in positive work behaviors and follow leaders’ instructions to complete work tasks efficiently. Lower leadership ability will lead to a crisis of trust, and employees may not follow the leaders’ instructions precisely, become less efficient, or even engage in CWB not beneficial to the work or the organization. Thus, the following hypothesis is proposed:

**Hypothesis 3 (H_3_):** The state of leadership ability characteristics impacts subordinate CWB.

#### The Mediation Role of Work Resources and Personal Resources

Work resources are natural stimuli that help employees to achieve work goals and motivate personal growth and development (Bakker et al., 2003; Bakker and Demerouti, 2007). Fewer work resources trigger negative attitudes and performance behaviors (Pyszczynski et al., 2004), such as absenteeism (Bakker, 2005) and poor motivation (Wong et al., 2011). The negative effect of personal resources on CWB is supported by empirical studies. For example, self-efficacy and organizational support (Yang et al., 2018; Ge and Chen, 2019) negatively affect CWB, and the depletion of organizational self-esteem (Su and Lin, 2019) positively affects employees’ silent behavior in CWB.

Differences in the quantity and quality of resources invested in the interaction between leaders and employees lead to differences in the quality of their exchanges (Graen et al., 1982). The state of psychological characteristics will affect subordinates’ perceptions of resources (Bandura, 1997; Lopez et al., 2002). Leaders with positive psychological characteristics can better understand subordinates, establish better leader–member exchange relationships, and give subordinates a stronger sense of organizational identity and belonging. Therefore, subordinates will feel more in control of their environment, which is equivalent to gaining personal resources (Hobfoll, 2002). Such leaders also voice more support for their subordinates, give them more freedom at work, and ensure a fairer distribution to run teams in an orderly fashion. In other words, they positively influence the work resources of their subordinates. As confident leaders provide more support for their employees (Bandura, 1997), their subordinates will perceive richer resources (work resources and personal resources). In the case of negative psychological characteristics, leaders appropriate resources for their benefit, or allocate resources to “insiders” and deprive other employees of resources. According to the COR theory, when individuals suffer from resource deficiency, they resort to CWB to regain resources or retaliate (Hobfoll, 1989). Thus, it can be hypothesized that resources may be the psychological mechanism by which psychological characteristics trigger subordinate CWB. The following hypotheses are thus proposed:

**Hypothesis (H_4a_):** The state of leadership psychological characteristics impacts subordinates’ work resources.
**Hypothesis (H_4b_):** The state of leadership psychological characteristics impacts subordinates’ personal resources.
**Hypothesis (H_5a_):** Work resources play a mediating role in the influence of leadership psychological characteristics on subordinate CWB.
**Hypothesis (H_5b_):** Personal resources play a mediating role in the influence of leadership psychological characteristics on subordinate CWB.

Counterproductive work behavior in organizational members may cause victims to experience a depletion in work resources, such as unfair resource allocation, limited career growth and development opportunities, and less job security. Subsequently, there can be a series of negative work behaviors, such as tardiness, disengagement, and unethical behaviors (Robinson et al., 2014). Counterproductive work behavior also depletes victims’ personal resources, attracts negative evaluations, sarcasm, hostility, and derogatory language, and lowers the victim’s self-evaluation (Huang et al., 2016; Ma et al., 2016). When personal resources are depleted but not replenished in time, ethical awareness decreases and CWB increases, signifying that individuals in a state of self-depletion are highly likely to consciously or unconsciously commit CWB due to a perceived loss of control (Baumeister et al., 2006; Gino et al., 2011; Tillman et al., 2015; Wang et al., 2017). When high-ranking leaders with more resources behave negatively, they trigger negative emotions in subordinates, reduce organizational identity, and deplete employees’ personal resources. In addition, subordinates’ expectations of fairness and developmental prospects are curtailed, and their work resources deplete. Thus, the more positive the leadership behavioral characteristics, the richer the resources perceived by subordinates. According to COR theory, when individuals suffer from resource loss, they resort to CWB to regain resources or retaliate. Thus, resources may be the psychological mechanism by which leadership behavioral characteristics trigger subordinate CWB. The following hypotheses are proposed:

**Hypothesis (H_6a_):** The state of leadership behavioral characteristics impacts subordinates’ work resources.
**Hypothesis (H_6b_):** The state of leadership behavioral characteristics impacts subordinates’ personal resources.
**Hypothesis (H_7a_):** Work resources play a mediating role in the influence of leadership behavioral characteristics on subordinate CWB.
**Hypothesis (H_7b_):** Personal resources play a mediating role in the influence of leadership behavioral characteristics on subordinate CWB.

Research shows that a company’s resources, rather than its products, are the factors that influence its performance development (Penrose, 1959). The heterogeneity of a company’s resources determines differences in competitiveness. Resources will only serve as the basis for competitive advantage when they are scarce, valuable, difficult to imitate, irreplaceable, and properly allocated (Barney, 1991). Leadership ability characteristics influence a company’s resource allocation. When leaders are extraordinary and highly capable of strategic decision-making, coordination, talent selection, communication, comprehensive analysis, and authorization, they are more likely to allocate resources rationally in the company. Subordinates will receive support from their leaders and the organization, find their position in the organization, improve their job satisfaction and self-efficacy, and subsequently implement a series of behaviors that meet the requirements of the leaders and are beneficial to the organization. Effective leaders strive to complete the work and reduce the occurrence of CWB to run the organization smoothly and effectively. However, a lack of the required ability characteristics will lead to poor resource allocation, which will weaken the competitive advantage of the enterprise and make it difficult to run the organization smoothly. Employees will be less motivated to work because of injustices in resource allocation and the incompetence of the leaders, and even exhibit CWB, which is detrimental to the organization’s interests. We propose the following hypotheses:

**Hypothesis (H_8a_):** The state of leadership ability characteristics impacts subordinates’ work resources.
**Hypothesis (H_8b_):** The state of leadership ability characteristics impacts subordinates’ personal resources.
**Hypothesis (H_9a_):** Work resources play a mediating role in the influence of leadership ability characteristics on subordinate CWB.
**Hypothesis (H_9b_):** Personal resources play a mediating role in the influence of leadership ability characteristics on subordinate CWB.

**FIGURE 2:**
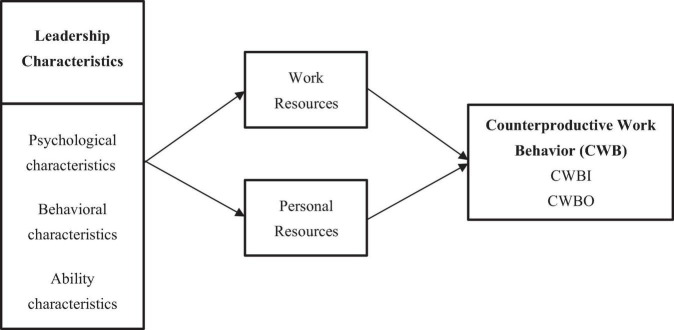
Theoretical model.

Based on the hypotheses and analysis, a theoretical model was developed, as shown in Figure 2.

### Methodology

#### Measures

##### Leadership Characteristics

The results of factor analysis of the initial scale of the multi-dimensional leadership characteristics are shown in Table 2. Most of the items have factor loadings greater than 0.5, 2 items have cross-loading, and 3 items have factor loadings less than 0.5; therefore, they are excluded. “Analyzing the excluded items, according to the results of corrected item-total correlation analysis, demonstrated that the reliability of the scale is good.” However, considering the length of the questionnaire that is required for the field survey, the scale needed to be simplified. The four items with the highest factor loadings were retained for each factor for a total of 12 items. Three factors cumulatively explained 67.24% of the variance, and the factor loading was greater than 0.5, indicating that the scale had good validity. The corrected item-total correlation value of all the questions was greater than 0.3, the conceptual Cronbach’s α coefficient was 0.910, and the dimensional Cronbach’ s α coefficients were 0.860, 0.819, and 0.800, for psychology, behavior, and ability, respectively, indicating that the scale had good reliability. The results are shown in Table 3.

**TABLE 2:** Factor analysis of the initial scale of leadership’s multi-dimensional characteristics.

Item	Factor 1	Factor 2	Factor 3
The leader is virtuous	**0.788**	0.254	0.242
The leader is upright	**0.746**	0.292	0.240
The leader plays fair	**0.632**	0.426	0.323
The leader is optimistic	**0.618**	0.329	0.182
The leader is kind to people	**0.552**	0.391	0.364
*The leader has better affinity*	***0.546***	***0.514***	*0.338*
*The leader can understand our difficulties*	*0.487*	*0.339*	*0.248*
*The leader does not put their own interests first but the interests of the team first*	*0.466*	*0.261*	*0.250*
The leader can find and point out subordinates’ mistakes at work in time	0.240	**0.727**	0.033
The leader is strong in management	0.314	**0.688**	0.268
The leader is a team player	0.252	**0.652**	0.374
The leader is able to help subordinates in terms of professional skills	0.139	**0.640**	0.292
The leader by example when leading	0.431	**0.611**	0.280
The leader is willing and proactively communicate with subordinates	0.481	**0.556**	0.192
The leader understands all the skills required by the department	0.407	**0.511**	0.230
The leader can value and listen to the opinions of subordinates	0.446	**0.510**	0.268
The leader takes the lead to give certain subordinate the cold shoulder	0.164	0.209	**0.734**
The leader scolds a subordinate in public whenever the subordinate fails to do to his/her satisfaction	0.106	0.248	**0.705**
The leader always takes advantage of his/her powers to bullies others	0.349	0.349	**0.696**
The leader is always very critical of certain subordinates	0.284	0.275	**0.657**
The leader always does things that take advantage of the company	0.433	0.087	**0.607**
*The leader has their own small circles and form cliques*	***0.515***	*0.011*	***0.604***
The leader is mean and small things become big	0.241	0.446	**0.578**
*The leader have a correct attitude*	*0.356*	*0.473*	*0.482*
***R* ^2^**	**20.48%**	**20.05%**	**18.39%**
			

***R^2^** represents Variation explained by each factor. The figure in bold type represents a factor load value greater than 0.5.*

**TABLE 3:** Reliability and validity analysis.

Leadership characteristics	Item	CITC	Dimensional Cronbach’s α	Conceptual Cronbach’s α	Loading	Explained variance
Psychological	The leader is virtuous	0.703	0.860	0.910	0.829	23.57%
	The leader is upright	0.697			0.807	
	The leader is optimistic	0.605			0.700	
	The leader is fair	0.745			0.638	
Behavioral	The leader scolds a subordinate in public whenever the subordinate fails to meet their satisfaction	0.552	0.819		0.810	23.35%
	The leader is always very critical of certain subordinates	0.643			0.734	
	The leader always takes advantage of his/her powers to bullies others	0.734			0.698	
	The leader takes the lead to give certain subordinate the cold shoulder.	0.586			0.682	
Ability	The leader can find and point out subordinates’ mistakes at work in time	0.503	0.800		0.797	20.32%
	The leader is strong in management	0.688			0.699	
	The leader is able to help subordinates in terms of professional skills	0.577			0.651	
	The leader is a team player	0.696			0.640	

*“psychological,” “behavioral,” and “ability” refer to leadership psychological characteristics, leadership behavioral characteristics, and leadership ability characteristics, respectively. CITC, corrected item–total correlation.*

##### Work and Personal Resources

For work resources, the scales of Bakker and Demerouti (2007) and Moorman (1991) were used for reference and integration. For personal resources, the scales used by Pierce et al. (1989); Scheier et al. (1994) and Schwarzer and Jerusalem (1995) were used as a reference according to Hobfoll’s (2002) classification. A seven-point Likert scale was used for scoring.

There were 19 and 25 questions for work and personal resources, respectively. Considering that the questionnaire needed to be distributed on site in the company, which consumes working hours, the scale needed to be simplified for data validity. According to the dimension division of work and personal resources in previous literature, 5 questions are extracted for each variable, which can not only satisfy the interpretation of the connotation of the variable, but also cover all the dimensions of the variable and improve the items’ language and description. Of the 211 questionnaires that were distributed on site in the company during working hours, and 198 valid questionnaires were obtained.

Reliability and validity tests and exploratory factor analyses were carried out. The results of the factor analysis showed that both work resources and personal resources were unidimensional scales, and that the work resource scale cumulatively explained 54.17% of the variance. All factor loadings were greater than 0.5, and the conceptual Cronbach’s α was 0.784. The personal resource scale cumulatively explained 50.85% of the variance; all the factor loadings were greater than 0.5 and the conceptual Cronbach’s α was 0.757, indicating that the scale had good reliability and validity. The results of the exploratory factor analysis and reliability test are shown in Table 4.

**TABLE 4:** Reliability and validity analysis.

	Item	CITC	Cronbach’s α	Loading	Explained variance
Work resources	My job helps me to develop and improve myself.	0.641	0.784	0.804	54.17%
	I do not see any development in my work.	0.624		0.794	
	I have greater autonomy in making decisions at work.	0.430		0.756	
	I am supported by workmates at work.	0.529		0.704	
	I am paid fairly compared to other workmates.	0.587		0.603	
Personal resources	I am always optimistic about my future.	0.605	0.757	0.783	50.85%
	I am optimistic about my status.	0.585		0.767	
	My presence is valued by the company.	0.529		0.717	
	I feel that I am in control of my life.	0.495		0.684	
	I am able to solve most problems or difficulties I encounter.	0.412		0.600	

*CITC: corrected item–total correlation.*

##### Counterproductive Work Behavior

The Bennett and Robinson (2000) scales were used as a reference for CWB. Items were adapted according to China’s national context and the current situation of enterprises, with five questions arranged for interpersonal CWB(CWBI) and organizational CWB (CWBO). The seven-point Likert scale was used for scoring (e.g., “I make fun of others at work”).

#### Participants and Procedure

The research was conducted through the offline distribution and recovery of paper questionnaires. To collect the questionnaire, we contacted some manufacturing enterprises and selected eight companies with the highest cooperation intention to participate in this study. The head of each company called the employees to a large conference room and asked them to complete the questionnaires. During the process of filling in the questionnaire, we supervise the employees on site to ensure that they do not interfere with each other. Once they completed the questionnaires, the employees were permitted to leave after placing the completed questionnaire on the table by the door.

A total of 268 questionnaires were received from eight companies. The interviewees represented a variety of positions, including front-line production employees and sales staff. After collecting the questionnaires, the quality of each questionnaire was checked and samples with a lot of missing data were deleted, along with samples that failed the reverse test, and those that had consistent or overly regular answers. The final count for valid questionnaires was 187 and the effective rate was 69.8%. In terms of gender, men accounted for 70.1%, women accounted for 29.9%, and the average age was 36.79 years. General staff accounted for 77% of the respondents and grassroots managers accounted for 13.4%.

### Results

#### Data Quality Test

The results of the analysis show that the average variance extracted values of the seven measurement variables: leadership psychological characteristics, leadership behavior characteristics, leadership ability characteristics, work resources, personal resources, CWBI and CWBO in the research model were 0.592, 0.454, 0.516, 0.362, 0.521, 0.512, and 0.486, respectively, and the composite reliability values were 0.850, 0.767, 0.808, 0.690, 0.842, 0.753, and 0.791, respectively, indicating that the convergent validity was acceptable.

Confirmatory factor analysis was used to test the discriminant validity of 7 variables in this study, and the results showed that the data fitting of the seven-factor model had a good fit (Table 5). All items were imported into SPSS for the common method variance test, and the variance explained by the first factor was 13.24%, which was less than 40%. More than one factor proved that the common method variance was within the acceptable range.

**TABLE 5:** Confirmatory factor analysis results.

Model	Factor	χ^2^/df	RMSEA	IFI	CFI
Seven-factor model	A_、_B_、_C_、_D_、_E_、_F_、_G	2.000	0.073	0.885	0.882
Six-factor model	A + B_、_C_、_D_、_E_、_F_、_G	2.465	0.088	0.827	0.825
five-factor model	A + B + C_、_D_、_E_、_F_、_G	2.620	0.093	0.807	0.804
Four-factor model	A + B + C_、_D_、_E_、_F + G	2.758	0.096	0.787	0.784
Three-factor model	A + B + C_、_D + E_、_F + G	2.782	0.097	0.782	0.779
Two-factor model	A + B + C + D + E_、_F + G	3.478	0.115	0.694	0.691
Single-factor model	A + B + C + D + E + F + G	4.330	0.133	0.588	0.584

*A, B, C, D, E, F, G respectively represent leadership psychological characteristics, leadership behavioral characteristics, and leadership ability characteristics, work resources, personal resources, CWBI and CWBO.*

#### Correlation Analysis

As shown in Table 6, all variables other than CWBO, which were not correlated with leadership behavioral characteristics, were pair-wise correlated.

**TABLE 6:** Correlation matrix between variables.

No.	Variable	M	SD	1	2	3	4	5	6	7
1	Psychology	6.36	0.66	(0.840)						
2	Behavior	6.40	0.73	0.402***	(0.745)					
3	Ability	6.25	0.68	0.527***	0.459***	(0.797)				
4	Work resources	5.77	0.86	0.457***	0.235**	0.565***	(0.664)			
5	Personal resources	5.98	0.79	0.362***	0.199**	0.589***	0.668***	(0.835)		
6	CWBI	1.26	0.53	−0.197**	−0.185*	−0.198**	−0.220**	−0.159*	(0.730)	
7	CWBO	1.23	0.46	−0.199**	−0.043	−0.240**	−0.237**	−0.223**	0.572**	(0.753)

*p < 0.05, **p < 0.01, ***p < 0.001. The brackets indicate the reliability of the variable scale.

#### Hypothesis Testing

##### Influence of Leadership Characteristics on Subordinates’ Counterproductive Work Behavior

The direct effects of leadership psychological characteristics, leadership behavioral characteristics, and leadership ability characteristics on CWB were tested. As shown in Table 7, according to M_10_ and M_14_, leadership psychological characteristics negatively affected CWBI and CWBO (*B* = −*0.17, p* < *0.01; B* = −*0.15, p* < *0.01*); according to M_11_, leadership behavioral characteristics negatively affected CWBI (*B* = −*0.16, p* < *0.01*); and according to M_12_ and M_15_, leadership ability characteristics negatively affected CWBI and CWBO (*B* = −*0.15, p* < *0.01; B* = −*0.16, p* < *0.01*). Hypotheses H_1_, H_2_, and H_3_ were therefore supported.

**TABLE 7:** Regression analysis of leadership characteristics vs. subordinates’ counterproductive work behavior (CWB), work resources, and personal resources.

	Work Resources				Personal Resources				CWBI				CWBO		
	M_1_	M_2_	M_3_	M_4_	M_5_	M_6_	M_7_	M_8_	M_9_	M_10_	M_11_	M_12_	M_13_	M_14_	M_15_
Constant	6.25***	2.30**	4.19***	1.58**	6.62***	3.81***	5.10***	2.28***	1.50***	2.66***	2.59***	2.50***	1.29***	2.27***	2.36***
Gender	−0.24	−0.18	−0.26	−0.19	−0.30*	−0.26*	−0.31*	−0.25*	−0.01	−0.03	0.00	−0.02	0.00	−0.02	−0.02
Age	−0.01	0.00	−0.00	-0.01	0.00	0.00	−0.00	0.00	−0.01	−0.01	−0.01*	−0.01	0.00	0.00	-0.00
Position	0.10	0.10	0.13	0.16*	−0.06	−0.05	0.03	0.00	0.02	0.02	0.00	0.00	0.01	0.00	-0.01
Psychology		0.58***				0.42***				−0.17**				−0.15**	
Behavior			0.29**				0.22**				−0.16**				
Ability				0.72***				0.67***				−0.15**			−0.16**
R^2^	0.03	0.23	0.09	0.36	0.03	0.15	0.07	0.37	0.015	0.06	0.059	0.054	0.001	0.043	0.060
△R^2^		0.20	0.06	0.32		0.12	0.04	0.34		0.045	0.044	0.040		0.042	0.058
F	2.12	13.50***	4.61**	25.31***	2.15^※^	8.25***	3.57**	27.04***	0.906	2.884*	2.845*	2.621*	0.073	2.044^※^	2.879*

^※^p < 0.1, * p < 0.05, **p < 0.01, ***p < 0.001.

##### Mediating Effects of Work and Personal Resources

As shown in Table 7, M_2_, M_3_, and M_4_, leadership psychological characteristics (*B* = *0.58, p* < *0.001*), leadership behavioral characteristics (*B* = *0.29, p* < *0.01*), and leadership ability characteristics (*B* = *0.72, p* < *0.001*) had a significant positive effect on work resources, thus supporting hypotheses H_4a_, H_6a_, and H_8a_. According to M_6_, M_7_, and M_8_, leadership psychological characteristics (*B* = *0.42, p* < *0.001*), leadership behavioral characteristics (*B* = *0.22, p* < *0.01*), and leadership ability characteristics (*B* = *0.67, p* < *0.001*) had a significant positive effect on personal resources. These results supported hypotheses H_4b_, H_6b_, and H_8b_.

According to M_2_, M_3_, M_14_, and M_15_ in Table 8, work resources and personal resources had a significant negative effect on both subordinates’ CWBI (*B* = −*0.15, p* < *0.01; B* = −*0.12, p* < *0.05*) and subordinates’ CWBO (*B* = −*0.14, p* < *0.01; B* = −*0.14, p* < *0.001*).

**TABLE 8:** Analysis of the mediating effects of work resources and personal resources.

	CWBI				CWBO																
	M_1_	M_2_	M_3_	M_4_	M_5_	M_6_	M_7_	M_8_	M_9_	M_10_	M_11_	M_12_	M_13_	M_14_	M_15_	M_16_	M_17_	M_18_	M_19_	M_20_	M_21_
Constant	1.50***	2.42***	2.28***	2.66***	2.91***	2.95***	2.59***	3.11***	3.06***	2.50***	2.68***	2.63***	1.29***	2.13***	2.21***	2.27***	2.52***	2.69***	2.36***	2.49***	2.54***
Gender	−0.01	−0.05	−0.05	−0.03	−0.05	−0.05	−0.00	−0.03	−0.03	−0.02	−0.05	−0.04	0.00	−0.04	−0.05	−0.02	−0.04	−0.05	−0.02	−0.03	−0.04
Age	−0.01	−0.01^※^	−0.01^※^	−0.01	−0.01	−0.01	−0.01*	−0.01*	−0.01*	−0.01^※^	−0.01^※^	−0.01^※^	0.00	−0.00	−0.00	0.00	−0.00	−0.00	−0.00	−0.00	−0.00
Position	0.02	0.03	0.01	0.02	0.03	0.01	−0.00	0.01	−0.02	0.00	0.02	0.00	0.01	0.02	−0.00	0.00	0.02	−0.00	−0.01	0.01	−0.01
Psychology				−0.17**	−0.11	−0.14*											−0.15**	−0.08	−0.10^※^			
Behavior							−0.16***	−0.12*	−0.14*													
Ability										−0.15**	−0.07	−0.12^※^							−0.16**	−0.10^※^	−0.11^※^
Work resources		−0.15**			−0.11*			−0.12**			−0.11*				−0.14**			−0.11*			−0.09^※^	
Personal resources			−0.12*			−0.08			−0.09^※^			−0.06			−0.14**			−0.11*			−0.08
R^2^	0.015	0.07	0.04	0.06	0.09	0.07	0.06	0.10	0.08	0.05	0.08	0.06	0.001	0.06	0.05	0.04	0.07	0.07	0.06	0.07	0.07
△R^2^		0.06	0.03	0.05	0.03	0.01	0.04	0.04	0.02	0.04	0.02	0.00		0.06	0.05	0.04	0.03	0.03	0.06	0.02	0.01
F	0.906	3.49**	2.10^※^	2.88*	3.37**	2.74*	2.85*	3.84**	2.99*	2.62*	3.02*	2.27*	0.073	2.98*	2.59*	2.04^※^	2.830*	2.782*	2.88*	2.99*	2.78*

^※^p < 0.1, * p < 0.05, **p < 0.01, ***p < 0.001.

According to M_5_ and M_6_, after work resources were entered into the regression equation, the effect of leadership psychological characteristics on subordinates’ CWBI was no longer significant (*B* = −*0.11, p* > *0.05*), while the effect of work resources was significant (*B* = −*0.11, p* < *0.05*), indicating that work resources fully mediated the effect of the state of leadership psychological characteristics on CWBI. After personal resources were entered into the equation, the direct effect of the state of leadership psychological characteristics on subordinates’ CWBI remained significant (*B* = −*0.14, p* < *0.05*), while the effect of personal resources was not significant (*B* = −*0.08, p* > *0.1*). Therefore, personal resources had no mediating effect. According to M_17_ and M_18_, for CWBO, the direct effect of leadership psychological characteristics was not significant (*B* = −*0.08, p* > *0.1*), while the effect of work resources was significant (*B* = −*0.11, p* < *0.05*) after work resources were entered into the regression equation. However, after personal resources were entered into the equation, the direct effect of leadership psychological characteristics was marginally significant (*B* = −*0.10, p* < *0.1*) and the effect of personal resources was significant (*B* = *−0.11, p* < *0.05*). Therefore, these two variables had a partial mediating effect. This result partially supported hypotheses H_5a_ and H_5b_.

According to M8 and M9, after work resources were entered into the regression equation, the effect of leadership behavioral characteristics on subordinates’ CWBI was significant (*B* = −*0.12, p* < *0.05*), the absolute value of the effect size decreased, and the effect of work resources was significant (*B* = −*0.12, p* < *0.01*); that is, work resources partially mediated the effect of the state of leadership behavioral characteristics on CWBI. After personal resources were entered into the equation, the direct effect of leadership psychological characteristics on subordinates’ CWBI was significant (*B* = −*0.14, p* < *0.05*), the absolute value of the effect size decreased, and the effect of personal resources was marginally significant (*B* = −*0.09, p* = *0.065*). Therefore, personal resources had a partial mediating effect. These results partially supported hypotheses H_7a_ and H_7b_.

According to M_11_ and M_12_, after work resources were entered into the regression equation, the effect of leadership ability characteristics on subordinates’ CWBI was no longer significant (*B* = −*0.07, p* > *0.1*), while the effect of work resources was significant (*B* = −*0.11, p* < *0.05*), indicating that work resources fully mediated the effect of leadership ability characteristics on CWBI. However, after personal resources were entered into the equation, the direct effect of leadership ability characteristics on subordinates’ CWBI was marginally significant (*B* = −*0.12, p* < *0.1*) and the effect of personal resources was not significant (*B* = −*0.06, p* > *0.1*). Therefore, personal resources had no mediating effect. According to M_20_ and M_21_, for CWBO, the direct effect of leadership ability characteristics was marginally significant (*B* = −*0.10, p* = *0.09*), and the effect of work resources was also marginally significant (*B* = −*0.09, p* = *0.07*), after work resources were entered into the equation. However, after personal resources were entered into the equation, the direct effect of leadership ability characteristics was marginally significant (*B* = −*0.11, p* = *0.07*), and the effect of personal resources was not significant (*B* = −*0.08, p* > *0.1*). Therefore, work resources and personal resources had no mediating effect, and the results supported hypothesis H_9a_, but not hypothesis H_9b_.

#### Strengths Analysis

To compare the magnitude of the effects of the three leadership characteristics on subordinates’ CWB, a strengths analysis was conducted using the approach of Shi and Li (2005) as a reference. The results are shown in Table 9.

**TABLE 9:** Strength analysis of the three leadership characteristics.

	Dependent variable: CWBI				Dependent variable: CWBO			
	R^2^	X1	X2	X3	R^2^	X1	X2	X3
	0	0.039	0.034	0.039	0	0.040	0.002	0.058
X1	0.039	-	0.013	0.012	0.040	-	0.002	0.026
X2	0.034	0.018	-	0.016	0.002	0.039	-	0.062
X3	0.039	0.012	0.011	-	0.058	0.007	0.006	-
X1 + X2	0.052	-	-	0.006	0.041	-	-	0.033
X1 + X3	0.051	-	0.007	-	0.065	-	0.009	-
X2 + X3	0.051	0.008	-	-	0.064	0.011	-	-
X1 + X2 + X3	0.058	-	-	-	0.074	-	-	-
Decomposition of R^2^		0.021	0.018	0.020		0.025	0.005	0.045
Percent in estimated variance of variables whose advantage is to be compared		35.62%	30.46%	33.91%		33.33%	6.76%	60.81%
								

*X1, X2, and X3 represent leadership psychological characteristics, leadership behavioral characteristics, and leadership ability characteristics, respectively.*

In terms of the effect of leadership characteristics on subordinates’ CWBI, the three leadership characteristics did not differ significantly. Meanwhile, in terms of the effect of leadership characteristics on subordinates’ CWBO, the effect of leadership ability characteristics was significantly greater than the other two characteristics. Subordinates would explicitly do something specific to the employer only when leadership ability characteristics were insufficient and detrimental to subordinates’ work resources. This may have something to do with whether subordinates treat leaders as representatives of the employer. In most scenarios, leaders represented the employer. Leadership’s psychology and behavior characteristics were personal and had nothing to do with the employer. Leadership ability characteristics were highly related in work and employers. When leadership ability characteristics were insufficient, subordinates had a lower degree of recognition toward the employer, and their sense of organizational identification decreased, giving rise to CWBO.

Chinese traditions and collectivist beliefs have changed due to the influence of Western thought. Chinese people today no longer follow authority figures blindly but value the hierarchy of status (Farh et al., 1997). Employees’ attitude toward work is no longer single-minded, and the turnover rate has increased significantly. In this context, when leaders have negative psychological characteristics, such as bossiness and lack of moral character, or negative behavioral characteristics, such as bullying and being critical of certain workers, subordinates will resign and find another job instead of choosing CWBO. However, when subordinates’ loss of resources is caused by a lack of leadership ability, they will choose CWBO because they are unwilling to give up the employer’s benefits and environment, but they also need to relieve the pressure caused by the loss of work resources.

## General Discussion

Based on an analysis of the literature, we found that leaders play an important role in research on organizational employee behaviors (e.g., CWB, organizational citizenship behaviors, extra-role behaviors, innovation behaviors, and unethical pro-organizational behaviors). However, research on leaders has not yet developed a formal structure. More attention has been paid to leadership styles and leadership behaviors. This study used grounded theory and summarized leadership factors influencing subordinates’ CWB as leadership characteristics, and proposed three dimensions: leadership psychological characteristics, leadership behavioral characteristics, and leadership ability characteristics. From leaders’ point of view, this study provides new thinking and new perspectives for the study of employee behaviors.

The ecological validity of the theoretical model proposed in this paper is proven through empirical research and the theoretical model has been established. In terms of the influence of leadership behaviors on subordinates’ behaviors, previous studies have found that leaders’ CWB can be transmitted from top to bottom through an employer’s vertical management hierarchy, ultimately giving rise to similar behavioral characteristics in subordinates (Fu and Deshpande, 2012; Mawritz et al., 2012; O’ Fallon and Butterfield, 2012; Robinson et al., 2014; Wang et al., 2020). This phenomenon has been confirmed in different cultures, including Spain (Ruiz et al., 2010) and China (Fu and Deshpande, 2012) and is consistent with the conclusions of this study. Leaders’ positive behavioral characteristics prompt subordinates to make positive behavioral responses, while leaders’ negative behavioral characteristics will trigger negative behavioral responses, and subordinates will further implement CWB to the employer or other employees.

In terms of the influence of leadership ability on subordinates’ behaviors, leadership ability is the foundation and guarantee of leadership because leaders are the core personnel of an organization. Leadership ability is also a necessary condition for leaders to build trust among employees. The level of leadership ability affects observers’ psychological cognition toward leaders and further affects their behavior (Boyce et al., 2010; Zhou and Long, 2016). Leadership ability significantly and positively predicts individual employees’ performance behaviors (Borman, 1993; Conway, 1999). This result is consistent with the conclusions of the present study. The higher a leader’s leadership ability, the more employees will trust the leader and the more employees will implement positive work behaviors and complete their work tasks efficiently and according to the leader’s instructions. On the contrary, employees’ trust in the leader will decrease and they are more likely to not follow the leader’s instructions, display low levels of productivity, or even resort to CWB. According to social identity theory, if leaders win the trust of employees, employees will have a sense of belonging and an emotional attachment to the employer, view themselves as “insiders” (Wang and Kim, 2013), and have a sense of group identity. This will give rise to organizational citizenship behaviors (Tajfel, 1978).

With regard to the influence of leadership psychological characteristics on subordinates’ behaviors, leaders with high emotional intelligence are able to understand employees’ feelings, enhance employees’ degree of satisfaction in their employer (Dasborough and Ashkanasy, 2002), pay more attention to creating an atmosphere of fairness, apply a gentler approach to resolving conflict and contradictions within the team, establish positive leader–member relationships, form a good team atmosphere (Daus and Ashkanasy, 2010), and arouse employees’ positive work attitudes and behaviors (Mayer et al., 2007). With high empathy competencies, leaders allow employees to identify themselves as “insiders” and thus stimulate subordinates’ positive emotions and perceptions to implement organizational citizenship behaviors (Liu et al., 2015). This is consistent with the conclusions of the present study. Therefore, leaders with positive psychological characteristics are sympathetic to employees, have trans-positional competencies, encourage employees to stay positive, and motivate them to work hard, and help them to improve their performance. If leaders behave badly, fail to match their words with their deeds, have little empathy, and do not pay attention to the commitments they make to employees, the quality of the relationship between leaders and subordinates will deteriorate, subordinates will harbor negative thoughts, work slower, or implement other CWBs.

According to the results of strengths analysis, for CWBI, there is little difference in the importance of the three leadership characteristics. However, for CWBO, the importance of leadership ability characteristics is significantly higher than for leadership psychological characteristics and leadership behavioral characteristics. In addition, the importance of work resources and personal resources differs in the influence of different leadership characteristics on subordinates’ CWB. According to COR theory, employees’ behaviors probably stem from a need to re-obtain or retain resources. Resources therefore play a mediating role. These two resources are similar in importance to the influence of leadership psychological characteristics on subordinates’ CWB. Leadership behavioral characteristics are significantly more inclined to influence subordinates’ CWB through personal resources, while leadership ability characteristics are slightly more inclined to act on subordinates’ CWB through work resources.

### Theoretical Contribution

Previous studies that introduced the COR theory into the field of CWB examined it from the viewpoint of the internal level of employees’ emotional resources, self-control resources, and cognition. However, this paper is closer to the actual organizational environment since it starts from the resources that employees can obtain from the organization rather than the psychological variables, as in previous studies. This is helpful to further verify the explanatory role of the COR theory in the field of CWB. Additionally, it provides new ideas for studying other behaviors that are affected by leadership in organizations and the organizational ethical climate.

This study contributes to literature on leadership. This study contributes to literature on leadership, by dividing leadership characteristics into psychological, behavioral and ability characteristics, and thereby, addressing the shortcomings of previous research on leadership. Additionally, it contributes to the field of leadership by analyzing and comparing different leadership characteristics. Previous studies have no theoretical and practical basis for the division of leadership characteristics, however, in this study it is based on a significant amount of grounded theory data and a rigorous analysis for an in-depth exploration, which can serve as a reference for future research.

### Limitation and Future Directions

In Study I, grounded theory was used to summarize the antecedent variable of CWB, “leadership factors,” as leadership characteristics, and three dimensions were determined. Although the process of coding and analysis was completed scientifically and accurately by several researchers, who consulted with each other, according to the steps adopted by Glaser and Strauss (2006) to develop the grounded theory, the analysis results are subject to the influence of researchers’ subjective experience. Therefore, future researchers may ask for help from other scholars in different fields or use qualitative analysis software to refine relevant concepts more objectively and accurately.

Mature foreign scales were used for most of the questionnaires. Most CWB scale development studies have been conducted in the context of European and American cultures. Cultural differences may have an impact on the measurement of questionnaires. Therefore, in future, localized and up-to-date CWB scales should be developed to enhance empirical research.

In this study, employees’ views on their leaders were measured and evaluated from their perspective. However, existing studies have shown that employees’ views on the relationship between employees and leaders are somewhat different from those of leaders. Thus, reflecting the interaction between employees and leaders from a single perspective, that is, from the perspective of employees, may not be comprehensive. Therefore, in future, tools such as HLM or Mplus, should be used to carry out multilevel analysis from the perspective of both leaders and employees.

#### Practical Implications

Since ancient times in China, there has been a saying that “Officials one rank superior crush the inferior”. Leaders have a special status in the enterprise, hold a high position, have resources and decision-making power, and their words and deeds will have an impact on employees. Therefore, exploring the influence on subordinates’ CWB from the perspective of leadership is an effective way to inhibit CWB. Leaders in different cultural groups have great differences in psychology and behavior, and their abilities are also uneven. Moreover, as the most unique and authoritative existence in each group, leaders’ psychology, behavior and ability will directly affect the behavior of subordinates. Therefore, starting from the characteristics of leadership, first, enterprises should evaluate and require the leadership’s ideological and moral level, psychological quality, attitude toward others and other psychological characteristics, and select excellent leaders from the root, so as to suppress the subordinates’ CWB. Second, cultivate the ability of leadership, including professional skills, management ability, work arrangement ability, strategic decision-making, interpersonal communication ability, etc., set a good example for subordinates, establish a good leader-member relationship with subordinates, so as to inhibit subordinates from CWB. Finally, restrain the behavior of leaders and encourage them to implement behaviors that are conducive to improving organizational performance and forming a healthy organizational atmosphere and culture, including taking the lead in completing work tasks, communicating with subordinates more, listening to their opinions and suggestions, and caring and helping more Subordinates, etc., make employees feel in a warm working atmosphere and reduce the implementation of CWB.

In addition, this paper provides a new manner through which companies can reduce the occurrence of CWB. Due to different characteristics of leaders will lead to different cognitive differences of employees on resources, and the cognitive differences of employees on resources will also lead to different behavioral choices. Therefore, starting from the theory of resource conservation, this paper links the resources that employees get from the organization with their behaviors, and attempts to avoid subordinates’ CWB from the perspective of resource allocation according to work.

## Data Availability Statement

The original contributions presented in the study are included in the article/supplementary material, further inquiries can be directed to the corresponding author/s.

## Ethics Statement

The studies involving human participants were reviewed and approved by the Ethics Committee of Tongji University. The participants provided their written informed consent to participate in this study.

## Author Contributions

YS led the conceptualization, wrote the original draft of the manuscript, carried out the formal analysis, and wrote – reviewed and edited the manuscript. XL supported the conceptualization, wrote the original draft of the manuscript, and wrote – reviewed, and edited the manuscript. Both authors contributed to the article and approved the submitted version.

## Conflict of Interest

The authors declare that the research was conducted in the absence of any commercial or financial relationships that could be construed as a potential conflict of interest.

## Publisher’s Note

All claims expressed in this article are solely those of the authors and do not necessarily represent those of their affiliated organizations, or those of the publisher, the editors and the reviewers. Any product that may be evaluated in this article, or claim that may be made by its manufacturer, is not guaranteed or endorsed by the publisher.
